# Transcription factors ERα and Sox2 have differing multiphasic DNA- and RNA-binding mechanisms

**DOI:** 10.1261/rna.080027.124

**Published:** 2024-08

**Authors:** Wayne O. Hemphill, Halley R. Steiner, Jackson R. Kominsky, Deborah S. Wuttke, Thomas R. Cech

**Affiliations:** 1 Department of Biochemistry, University of Colorado Boulder, Boulder, Colorado 80303, USA; 2 Howard Hughes Medical Institute and BioFrontiers Institute, University of Colorado Boulder, Boulder, Colorado 80303, USA

**Keywords:** RNA, DNA, transcription factor, kinetics

## Abstract

Many transcription factors (TFs) have been shown to bind RNA, leading to open questions regarding the mechanism(s) of this RNA binding and its role in regulating TF activities. Here, we use biophysical assays to interrogate the *k*_on_, *k*_off_, and *K*_d_ for DNA and RNA binding of two model human TFs, ERα and Sox2. Unexpectedly, we found that both proteins exhibit multiphasic nucleic acid–binding kinetics. We propose that Sox2 RNA and DNA multiphasic binding kinetics can be explained by a conventional model for sequential Sox2 monomer association and dissociation. In contrast, ERα nucleic acid binding exhibited biphasic dissociation paired with novel triphasic association behavior, in which two apparent binding transitions are separated by a 10–20 min “lag” phase depending on protein concentration. We considered several conventional models for the observed kinetic behavior, none of which adequately explained all the ERα nucleic acid–binding data. Instead, simulations with a model incorporating sequential ERα monomer association, ERα nucleic acid complex isomerization, and product “feedback” on isomerization rate recapitulated the general kinetic trends for both ERα DNA and RNA binding. Collectively, our findings reveal that Sox2 and ERα bind RNA and DNA with previously unappreciated multiphasic binding kinetics, and that their reaction mechanisms differ with ERα binding nucleic acids via a novel reaction mechanism.

## INTRODUCTION

The human genome encodes approximately1500 transcription factors (TFs) (Zhang et al. 2012; Wingender et al. 2013, 2015; Ignatieva et al. 2015), which direct cell-type specificity and gene expression programs by interacting with a multitude of binding partners (Spitz and Furlong 2012). TFs modulate transcription by using their DNA-binding domains (DBDs) to stably interact with DNA elements, such as promoters and enhancers, with sequence specificity, and subsequently recruit various coactivator and repressor proteins via their effector domains (Schwabe et al. 1993; Frietze and Farnham 2011). However, the site of transcription is immersed in more than DNA and protein—it is also crowded with RNA. Thousands of RNA species are produced at loci where TFs are bound, such as mRNAs, enhancer RNAs, promoter antisense RNAs, and chromatin-enriched long noncoding RNAs (Werner and Ruthenburg 2015; Yang et al. 2021; Han and Li 2022). Additionally, long noncoding RNAs transcribed distally, even kilobases away, are capable of engaging in long-range interactions with chromatin (Mishra and Kanduri 2019; Rinn and Chang 2020). The prevalence of RNA at chromatin begs the question of whether RNA plays a direct role in regulating TFs.

Numerous TFs have been shown to bind RNA (Khalil et al. 2009; Hudson and Ortlund 2014; Hendrickson et al. 2016; Parsonnet et al. 2019; Skalska et al. 2021; Oksuz et al. 2023). In many cases, the TF RNA-binding domains are adjacent to their DNA-binding domains (Oksuz et al. 2023). Estrogen receptor α (ERα) (Steiner et al. 2022) and sex-determining region Y box 2 (Sox2) (Holmes et al. 2020) are two such TFs that bind DNA and RNA competitively with tight affinities, suggesting potential biological relevance for the RNA-binding activity. ERα and Sox2 can therefore be used as model systems to study RNA regulation of TF activities. Although RNA–DNA competition experiments provide some useful information, a detailed investigation of the mechanism(s) and kinetics for TF-polynucleotide association and dissociation is critical for understanding how TFs could be regulated by RNA binding.

ERα is a ligand-activated TF, which functions as the nuclear receptor for estrogen, a hormone that dictates reproductive development (Björnström and Sjöberg 2005; Deroo and Korach 2006) (mouse studies reviewed in Hewitt and Korach 2018). Abnormal ERα signaling leads to a variety of diseases such as metabolic and cardiovascular disease, neurodegeneration, and inflammation (Jia et al. 2015). Additionally, ERα is aberrantly expressed in 80% of breast cancers, making it a recurrent therapeutic target (Alluri et al. 2014). ERα is a 595 amino acid polypeptide (∼66 kDa) comprised of six domains, including DNA-binding, ligand-binding, and transcriptional activation domains (Ponglikitmongkol et al. 1988; Hewitt and Korach 2018). Its DBD facilitates sequence-specific DNA binding to the palindromic estrogen response element (ERE) motif (GGTCAnnnTGACC) and binds as a dimer via its two zinc finger elements (Schwabe et al. 1993; Kuntz and Shapiro 1997; Helsen et al. 2012). The hinge region sits just C-terminal of the DBD, and recent work has demonstrated that part of the hinge region is critical for RNA binding (but dispensable for DNA binding). ERα uses a combination of the DBD and hinge elements to preferentially bind hairpin RNA (hRNA) with no apparent sequence specificity (Xu et al. 2021; Steiner et al. 2022). Although in vitro experiments indicate that ERα RNA and DNA binding are competitive, and ERα has been shown to interact with RNA in vivo (Nassa et al. 2019; Xu et al. 2021), the question of how RNA may regulate ERα–DNA interactions remains an active area of investigation (Steiner et al. 2022).

Sox2, a member of the SoxB1 TF family, regulates pluripotency in embryonic stem cells via expression of the pluripotency-associated TFs Oct4 and Nanog and via repression of lineage-specific genes (Avilion et al. 2003; Chew et al. 2005; Zhang and Cui 2014). Additionally, Sox2 is critical for differentiating pluripotent stem cells to neural progenitors and maintaining the properties of neural progenitor stem cells (Zhang and Cui 2014). In mice, deletion of Sox2 is embryonic lethal (Avilion et al. 2003), whereas knockout in adult mice leads to the loss of hippocampal neurogenesis (Favaro et al. 2009). In humans, mutations in Sox2 have been associated with eye defects such as bilateral anophthalmia and microphthalmia (Fantes et al. 2003; Chassaing et al. 2014), as well as cognitive abnormalities (Kelberman et al. 2006; Sisodiya et al. 2006). Functional Sox2 contains 317 amino acids partitioned into two key domains (Nowling et al. 2000). The Sox2 high mobility group (HMG) domain binds DNA in the minor groove and recognizes a species-specific sequence centered around four highly conserved nucleotides (CCCATTGTTC in humans) (Grosschedl et al. 1994; Weiss 2001; Hou et al. 2017; Yesudhas et al. 2017; Dodonova et al. 2020; Schaefer and Lengerke 2020).

Multiple studies have reported that Sox2 and related HMG domain proteins bind RNA functionally in cells (Tung et al. 2010). For example, in vivo studies have suggested that lncRNAs interact (Hamilton et al. 2023) with Sox2 to regulate its function in stem cell pluripotency (Ng et al. 2012). Sox2 has been found to modulate alternative mRNA splicing (Tung et al. 2010). Although a recent study urged caution in interpreting cross-linking and immunoprecipitation (CLIP) experiments because they can detect binding interactions that were not present in vivo (Guo et al. 2024), much of the evidence for Sox2–RNA interaction in vivo is CLIP-independent (Hamilton et al. 2023). Biochemical studies show that the Sox2 HMG domain preferentially binds hRNA with no apparent sequence specificity (Holmes et al. 2020). Like ERα, Sox2 HMG domain binding to RNA and DNA was found to be competitive (Holmes et al. 2020). However, another study suggested that a novel RNA-binding module C-terminal of the HMG domain also contributes to Sox2 RNA binding, and that Sox2 can stably bind RNA and DNA simultaneously (Hou et al. 2020).

To better understand the mechanisms of RNA and DNA binding on the Sox2- and ERα-binding surfaces, we used fluorescence polarization (FP) and surface plasmon resonance (SPR) to measure their RNA and DNA association and dissociation kinetics. In contrast to the expectation for a simple binding reaction, both TFs exhibited complex multiphasic association and dissociation kinetics from RNA and DNA. We evaluated several common models for multiphasic association and dissociation to describe the observed kinetics for the two TF interactions with RNA and DNA. These findings reveal a previously unappreciated level of complexity in the ERα and Sox2 interactions with nucleic acids, and they suggest that the two TFs achieve multiphasic kinetics through different mechanisms.

## RESULTS

### ERα_DBD-Ext_ and Sox2_HMG_ equilibrium ligand binding

**FIGURE 1.**
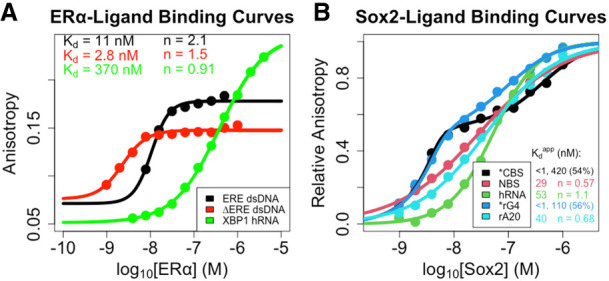
(*A*) ERα_DBD-Ext_- and (*B*) Sox2_HMG_-ligand-binding affinities. Equilibrium (end point) anisotropy values are plotted as function of protein concentration. Equilibrium data were fit to the Hill binding equation (Equation 1) or two-transition binding equation (Equation 2) via regression on linear axes to determine the apparent equilibrium dissociation constants ($K_{d}^{\mathrm{app}}$) and Hill coefficients (*n*). Values for $K_{d}^{\mathrm{app}}$ are in units of nanomolarity (nM). Dots are data points, and solid lines are regression fits. Data are from a single experiment for each ligand, and binding constant values are the average of independent experiments (one to three per interaction; see Table 1 for error analysis). We note that the ΔERE $K_{d}^{\mathrm{app}}$ is approximately half the ligand concentration for the assay, suggesting that the true *K*_d_ may be even lower, and that the apparent Hill coefficient (*n*) may be slightly inflated by the anomalous tight-binding curve. Thus, we make no assertion of positive cooperativity for the ERα_DBD-Ex_–ΔERE interaction. (*) Two-transition binding curves with $K_{d}^{\mathrm{app}}$ values shown alongside the proportion of the binding signal dynamic range attributable to the lower $K_{d}^{\mathrm{app}}$; no applicable Hill coefficient values.

ERα_180–280_, a region of the protein containing the canonical DBD and a set of basic residues from the hinge region (ERα_DBD-Ext_), and recombinant Sox2_40–123_, the region of the protein containing the HMG domain (Sox2_HMG_), were expressed and purified as previously described (Holmes et al. 2020; Steiner et al. 2022). We performed FP-based binding experiments with ERα_DBD-Ext_ and Sox2_HMG_ and a variety of dsDNA and RNA ligands to assess their binding affinities (Fig. 1) under the same experimental conditions we will use to measure binding kinetics.

For ERα_DBD-Ext_, we tested an 18 bp dsDNA containing its palindromic ERE recognition sequence (ERE dsDNA), a 15 bp dsDNA containing only half of its palindromic recognition sequence (ΔERE dsDNA), and a 37 nt hRNA derived from the X-box-binding protein 1 (XBP1) mRNA sequence (XBP1 hRNA) (Supplemental Fig. S1), based on previously established ERα RNA- and DNA-binding preferences (Steiner et al. 2022). We found that ERα_DBD-Ext_ bound ERE with high affinity ($K_{d}^{\mathrm{app}}$ ≈ 11 nM; see Table 1 for all error analyses) and positive cooperativity (*n* ≈ 2.1) (Fig. 1A; Table 1), whereas ERα_DBD-Ext_ bound ΔERE with comparable to higher affinity ($K_{d}^{\mathrm{app}}$ ≈ 2.8 nM) and less to no positive cooperativity (*n* ≈ 1.5) (Fig. 1A; Table 1). ERα_DBD-Ext_ bound the XBP1 hRNA with lower affinity ($K_{d}^{\mathrm{app}}$ ≈ 370 nM) and no apparent cooperativity (*n* ≈ 0.91) (Fig. 1A; Table 1). These findings are consistent with prior studies (Steiner et al. 2022). We note that the anisotropy dynamic range was less for the ΔERE versus ERE binding curve, consistent with a lower TF-DNA binding stoichiometry for ΔERE versus ERE, as expected from prior studies (Schwabe et al. 1993; Steiner et al. 2022).

**TABLE 1.** Kinetic constant values from FP-based ERα_DBD-Ext_- and Sox2_HMG_-binding experiments

Protein	Ligand	$K_{d}^{\text{app}}$ (nM)	*n* (Hill)	$k_{\mathrm{on}}^{\mathrm{app}}$ (M^−1^s^−1^)	$k_{\mathrm{off}\;\mathrm{fast}}^{\mathrm{app}}$ (×10^−2^ sec^−1^)	$k_{\mathrm{off}\;\mathrm{slow}}^{\mathrm{app}}$ (×10^−4^ sec^−1^)
ERα_DBD-Ext_	ERE dsDNA	11 ± 2.1	2.1 ± 0.30	5.8 ± 3.1 × 10^4^	4.7 (71%)	7.3 (29%)
	ΔERE dsDNA	2.8 ± 0.6^a^	1.5 ± 0.22^a^	8.7 ± 4.6 × 10^4^	8.9 ± 4.4 (60%)	5.9 ± 1.1 (40%)
	XBP1 hRNA	370	0.91	14 × 10^4^	5.6 ± 2.7 (64%)	6.6 ± 1.2 (36%)
Sox2_HMG_	CBS dsDNA	≤1 (54%), 420 ± 120^b^	n/a^b^	0.75 × 10^6^	5.0 ± 2.3 (67%)	13 ± 2.1 (33%)
	G4 RNA	≤1 (44%), 110 ± 32^b^	n/a^b^	1.1 × 10^6^	5.4 (73%)	9.6 (27%)
	NBS dsDNA	29 ± 9.1	0.57 ± 0.06	n.d.	n.d.	n.d.
	hRNA	53 ± 16	1.1 ± 0.21	n.d.	n.d.	n.d.
	poly(A) RNA	40 ± 13	0.68 ± 0.11	n.d.	n.d.	n.d.

**FIGURE 2.**
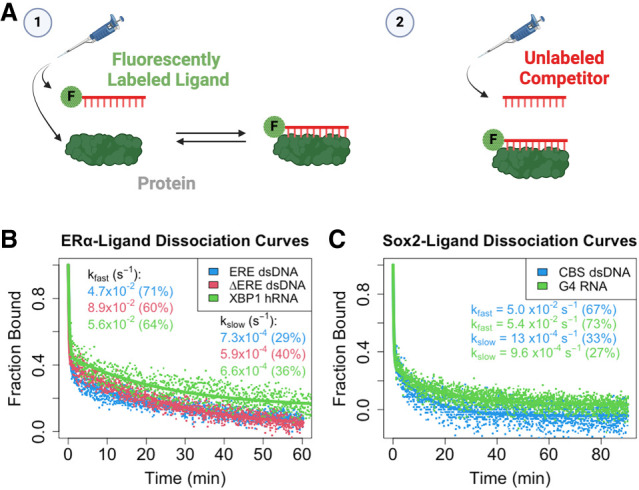
ERα_DBD-Ext_ and Sox2_HMG_ exhibit multiphasic ligand dissociation. (*A*) Graphical summary of FPCD experiments. (*1*) Fluorescently labeled polynucleotide is mixed with protein and incubated at 4°C for a variable amount of time, and then (*2*) an excess of unlabeled polynucleotide (i.e., competitor) is added to the protein–ligand reaction and polarization is monitored over time (at 4°C) to observe protein–ligand dissociation kinetics. (*B*,*C*) Dissociation curves from FPCD experiments. FPCD experiments (*A*) were performed using 5 nM ligand, 10 µM competitor, and 100–500 nM protein; protein–nucleic acid–binding reactions were incubated long enough to reach equilibrium before competitor addition. Anisotropy traces were normalized to the internal controls to give “Fraction Bound” over time, and then normalized data were fit with biexponential regression (Equation 5) to determine rate constants. Dots are data points and solid lines are regression fits from a single experiment for each ligand. Rate constants and (in parentheses) the percent contributions of fast versus slow components to the biexponential regression are reported with error in Table 1.

The table includes apparent equilibrium dissociation constants ($K_{d}^{\mathrm{app}}$) and Hill coefficients (*n*) from Figure 1 (fit with Equations 1 and 2), apparent initial association rate constants ($k_{\mathrm{on}}^{\mathrm{app}}$) from Figure 4, and apparent dissociation rate constants ($k_{\mathrm{off}}^{\mathrm{app}}$) from Figure 2 (fit with Equation 5). Values are mean ± ½ range (across two to three independent experiments). Values without error are based on a single experiment. For $K_{d}^{\mathrm{app}}$, values in parentheses are the percent signal contributions of the first transitions in two-transition binding regression, and for $k_{\mathrm{off}}^{\mathrm{app}}$, values in parentheses are the percent contributions of the fast or slow components in biexponential regression. Percentages are the averages across two to three independent experiments, or values from a single experiment if the associated rate constants have no error.

(n/a) Not applicable, (n.d.) not determined.

^a^$K_{d}^{\mathrm{app}}$ is close to ligand concentration; it is possible that *K*_*d*_ < $K_{d}^{\mathrm{app}}$ and *n* (Hill coefficient) are artificially inflated.

^b^Binding curves were fit with a two-transition equation, not the Hill equation.

For Sox2_HMG_, we tested a 10 bp dsDNA containing its cognate binding sequence (CBS dsDNA) (Holmes et al. 2020), and for comparison we also measured the binding affinities for a 19 bp dsDNA with a nonspecific binding sequence (NBS dsDNA), a 43 nt hRNA, a (G_3_A_2_)_4_ RNA that adopts a G-quadruplex (G4) structure (rG4), and a 20 nt poly(A) RNA (rA20) (Supplemental Fig. S1; Fig. 1B; Table 1), based on established Sox2 RNA-binding preferences (Holmes et al. 2020; Hamilton et al. 2022). Our findings indicate that Sox2_HMG_ binding to CBS dsDNA and G4 RNA were best described by a two-transition binding curve, whereas Sox2_HMG_ binding to NBS dsDNA, hRNA, and poly(A) RNA fit well to a standard Hill binding equation. The Sox2_HMG_ CBS and rG4 high-affinity binding transitions both had $K_{d}^{\mathrm{app}}$ ≤ 1 nM, being limited by the ligand concentration in our assays, whereas their lower-affinity binding transitions had $K_{d}^{\mathrm{app}}$ of 420 and 110 nM, respectively (see Table 1 for error analysis). For CBS dsDNA, the two transitions were previously attributed to Sox2_HMG_ initial sequence-specific binding followed by subsequent nonspecific binding (Holmes et al. 2020; Hamilton et al. 2022). Relative to the high-affinity binding transition, Sox2_HMG_ exhibited ≥30× greater affinity for CBS dsDNA and G4 RNA than for NBS dsDNA, hRNA, and poly(A) RNA ($K_{d}^{\mathrm{app}}$ ≈ 29–53 nM). We also note that the Sox2_HMG_ NBS dsDNA and poly(A) RNA-binding curves exhibited modest negative cooperativity (*n* ≈ 0.57–0.68), whereas Sox2_HMG_ bound the hRNA without apparent cooperativity (Fig. 1B; Table 1). All these findings are in agreement with prior studies (Holmes et al. 2020; Hamilton et al. 2022), validating the reagents and methods for the subsequent measurements below.

### ERα_DBD-Ext_ and Sox2_HMG_ ligand dissociation are multiphasic

We measured the ERα_DBD-Ext_- and Sox2_HMG_-ligand dissociation kinetics using FP-based competitive dissociation (FPCD) experiments. These involve preincubation of protein and fluorescently labeled nucleic acid followed by self-competition with unlabeled nucleic acid and observation of binding states by FP (Fig. 2A). For each protein, we tested their target DNA and highest affinity RNA as ligands. Contrary to the expectation for a simple binding scheme (i.e., protein + ligand ↔ protein-ligand), both ERα_DBD-Ext_ and Sox2_HMG_ exhibited multiphasic dissociation from all ligands that we tested (Fig. 2B,C). For ERα_DBD-Ext_, the ligand dissociation curves were well fit by biexponential regression and produced similar rate constants for all the nucleic acids tested. The rate constants and other relevant parameter values for these regressions are summarized in Table 1.

We then asked what could be producing the biphasic dissociation curves for our TF–nucleic acid interactions. Previously, we demonstrated that direct transfer is used by multiple nucleic acid–binding proteins to transfer between polynucleotide species through unstable ternary intermediates (Hemphill et al. 2023). Furthermore, at the competitor concentrations used in our FPCD experiments (Fig. 2), protein–polynucleotide dissociation might occur via both direct transfer and intrinsic dissociation in comparable proportions (Hemphill et al. 2023). If the fast components of the ERα_DBD-Ext_ biphasic dissociation curves were the result of ligand displacement via direct transfer, their dissociation curves should become monophasic slow in the absence of competitor. To test this hypothesis, we induced complex dissociation by dilution rather than competitor addition with FP-based jump dilution (FPJD) experiments.

**FIGURE 3.**
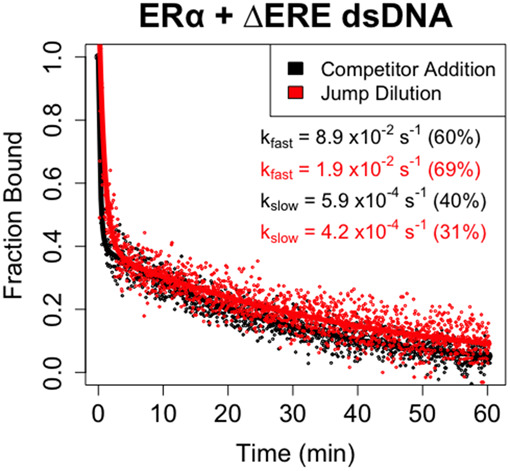
ERα_DBD-Ext_ multiphasic ligand dissociation is independent of competitor. Dissociation curves from FPCD versus FPJD experiments. FPJD experiments were performed for the ERα_DBD-Ext_–ΔERE interaction using 50 nM ligand and 50 nM protein (predilution), with protein–ligand reactions being incubated to equilibrium before dilution. The protein–polynucleotide reaction was then diluted ∼80-fold in buffer (at 4°C) and polarization was monitored over time postdilution (at 4°C) to quantify protein–ligand dissociation kinetics. FPCD experiments were performed as described in Figure 2. Anisotropy traces were normalized to the controls to give “Fraction Bound” over time, and then normalized data were fit with biexponential regression to determine rate constants. Dots are data points and solid lines are regression fits (Equation 5) from single experiments. Rate constants are the average values from all independent experiments (two for FPCD, three for FPJD), with percent contributions of fast and slow components to the biexponential curve in parentheses. Error analyses for FPCD values are in Table 1, and for FPJD *k*_fast_ = 1.9 ± 0.91 × 10^−2^ sec^−1^, *k*_slow_ = 4.2 ± 1.2 × 10^−4^ sec^−1^ (mean ± ½ range).

For ERα_DBD-Ext_, we measured ΔERE dsDNA dissociation because it had the binding properties most compatible with the limitations of FPJD methodology. Notably, ΔERE dissociation was still biphasic in the absence of competitor (Fig. 3), with no reduction in the contribution of the fast component to the biexponential regression (FPCD ≈ 60% ± 3.9%, FPJD ≈ 69% ± 6.1%). In essence, the biphasic nature of the dissociation curves was not attributable to direct transfer. However, the presence of competitor appeared to greatly (4×–5×) increase the rate of the fast component (FPCD_fast_ ≈ 8.9 ± 4.4 × 10^−2^ sec^−1^ vs. FPJD_fast_ ≈ 1.9 ± 0.91 × 10^−2^ sec^−1^), but not slow component (FPCD_slow_ ≈ 5.9 ± 1.1 × 10^−4^ sec^−1^ vs. FPJD_slow_ ≈ 4.2 ± 1.2 × 10^−4^ sec^−1^), of the biexponential regression. This suggests that the ERα_DBD-Ext_-ΔERE complex state associated with fast dissociation is susceptible to direct transfer, whereas the state associated with slow dissociation is not. For Sox2_HMG_, we are unable to make a similar assessment, because the FPJD assay produced less signal-to-noise relative to the FPCD assay, and the Sox2_HMG_–CBS interaction had a lower anisotropy dynamic range than the ERα_DBD-Ext_–ERE interaction. Thus, the Sox2_HMG_ dissociation curves were too noisy for reliable analysis.

Ruling out direct transfer as the origin of the biphasic kinetics, we moved on to a second hypothesis. A prior in vitro study demonstrated that the RNA-binding affinity of our ERα_DBD-Ext_ construct is facilitated by a basic 18 amino acid sequence (RMLKHKRQRDDGEGRGE) from the hinge region of the protein, and these residues do not significantly affect binding affinity for ERE dsDNA (Steiner et al. 2022). Thus, we assessed whether these additional nucleic acid–binding residues in the protein explain the biphasic nature of our ERα_DBD-Ext_ dsDNA dissociation kinetics by providing a lower-affinity alternative binding site. We therefore compared the dsDNA dissociation kinetics for the ERα_DBD-Ext_ construct to a construct lacking the basic hinge region residues (ERα_DBD_) by FPCD. Our findings indicated that ERα dsDNA dissociation kinetics were still biphasic with ERα_DBD_, ruling out these additional basic residues as the cause for biphasic dissociation (Supplemental Fig. S2).

### ERα_DBD-Ext_, but not Sox2_HMG_, exhibits multiphasic association to target DNA

**FIGURE 4.**
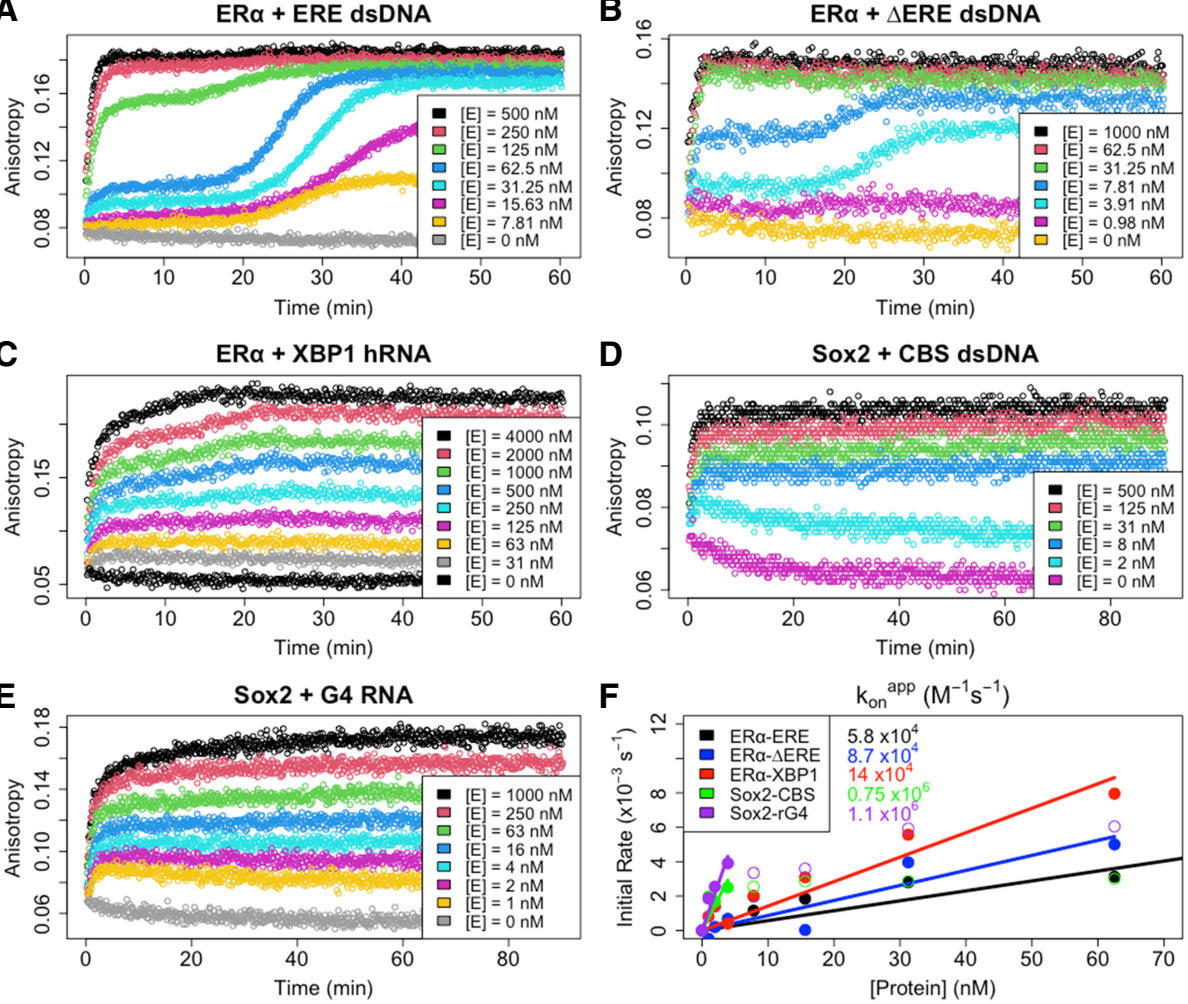
ERα_DBD-Ext_, but not Sox2_HMG_, exhibits multiphasic target DNA association. (*A*–*E*) FP-based association curves. Protein–ligand reactions were prepared after thermal equilibration (4°C), and anisotropy was monitored over time immediately after protein addition to quantify association kinetics. Ligand concentrations were 5 nM, and protein concentrations [E] are indicated. Data are from single representative experiments (of one to three per protein–ligand interaction). The first 10–45 min of association data were subjected to regression with an equation for monophasic (Equation 3) or biphasic (Equation 4) association; the regression fits are shown in Supplemental Figure S3. (*F*) Association rate constant analysis. Apparent initial association rates were determined with smoothing spline regression (see Materials and Methods) and are plotted as a function of protein concentration. Each interaction has an initial linear component, followed by a plateau in apparent association rate at higher protein concentrations, which corresponds to the incomplete association curves seen at higher protein concentrations because of methodological limitations. Apparent association rates from these linear stages were used for zero-intercept linear regression to calculate apparent association rate constants ($k_{\mathrm{on}}^{\mathrm{app}}$). Filled circles are data points used for linear regression, open circles are data points excluded from linear regression, and solid lines are linear regression fits. Rate constants are reported with any error in Table 1.

The observation of biphasic ligand dissociation kinetics implies the presence of multiple protein–ligand complexes. To probe this further, we performed FP-based ligand association experiments for ERα_DBD-Ext_ binding to ERE, ΔERE, and XBP1, and for Sox2_HMG_ binding to CBS and rG4 (Fig. 4A–E). Sox2_HMG_ binding to CBS dsDNA was strictly monophasic (Supplemental Fig. S3D). Sox2_HMG_ rG4 association appeared classically biphasic (i.e., fitting a biexponential) (Supplemental Fig. S3E), in which the second Sox2_HMG_ rG4 association phases in the biexponential regressions were ∼15× slower than the first phases. We note, however, that percent slow association phase contributions trended downward from ∼35% at 1 µM protein to <10% at 8 nM protein (Supplemental Fig. S3E), resulting in monophasic association at lower protein concentrations. This trend seemed to correlate to the second transition in the binding curve, and it suggests a monomer–dimer equilibrium (Fig. 1B).

In contrast, ERα_DBD-Ext_ exhibited multiphasic association for both DNA and RNA (Fig. 4A–C). For the dsDNA association, we observed a highly unusual triphasic association that was protein concentration–dependent. An approximately monophasic association phase was complete in ∼2 min (Supplemental Fig. S3A,B), followed by a 10–20 min “lag” phase, and ending in a second association phase that was complete on a timescale similar to that of the first association phase (Fig. 4A,B). Perplexingly, the “lag” phase was only evident at protein concentrations ≤10 × *K*_d_ of the DNA, whereas at higher concentrations ERα_DBD-Ext_ DNA association appeared monophasic (Fig. 4A,B). For ERα_DBD-Ext_ hRNA association, we observed what appeared to be biphasic association, but regression with a biexponential equation revealed an inadequate fit (Supplemental Fig. S3C). On closer inspection, the ERα_DBD-Ext_ hRNA association curves are more like the “transition” protein concentrations in the ERα_DBD-Ext_ dsDNA association curves (e.g., Fig. 4A, [*E*] = 125 nM), suggesting similar association mechanisms.

We used the first phases of the association curves to determine apparent initial association rate constants (Fig. 4F; Table 1). For ERα_DBD-Ext_, we find that initial nucleic acid association is ∼7000× to 17,000× slower than diffusion-limited binding (Table 1; Fersht 1985). For Sox2_HMG_, we find that CBS dsDNA and G4 RNA initial associations are likewise slower than diffusion-limited binding (∼1000× to 1300×) (Table 1). These rates are notably slower than some other TFs, suggesting a potential conformational barrier during initial ERα_DBD-Ext_ and Sox2_HMG_ nucleic acid–binding (Halford and Marko 2004).

To gain insights into the underlying ERα_DBD-Ext_ nucleic acid–binding mechanism(s), we compared our measured rate constants with the equilibrium binding data. We noted that ERα_DBD-Ext_ associates at a similar or modestly greater rate with hRNA versus dsDNA (Fig. 4F), despite having lower affinity and similar dissociation rates (Figs. 1A and 2B). Dividing the $k_{\mathrm{off}}^{\mathrm{app}}$ (apparent dissociation rate constants) by the $k_{\mathrm{on}}^{\mathrm{app}}$ for the respective ligands, which should yield the $K_{d}^{\mathrm{app}}$, suggests that the ERα_DBD-Ext_ dsDNA $K_{d}^{\mathrm{app}}$ is mostly influenced by the slow biexponential dissociation phase ($k_{\mathrm{off}\;\mathrm{slow}}^{\mathrm{app}}$/$k_{\mathrm{on}}^{\mathrm{app}}$; ERE = 13 nM, ΔERE = 6.8 nM, XBP1 = 4.7 nM), whereas the RNA $K_{d}^{\mathrm{app}}$ is mostly influenced by the fast biexponential dissociation phase ($k_{\mathrm{off}\;\mathrm{fast}}^{\mathrm{app}}$/$k_{\mathrm{on}}^{\mathrm{app}}$; ERE = 0.89 µM, ΔERE = 1.0 µM, XBP1 = 400 nM). We made similar comparisons for the Sox2_HMG_ CBS and rG4 interactions ($k_{\mathrm{off}\;\mathrm{slow}}^{\mathrm{app}}$/$k_{\mathrm{on}}^{\mathrm{app}}$ and $k_{\mathrm{off}\;\mathrm{fast}}^{\mathrm{app}}$/$k_{\mathrm{on}}^{\mathrm{app}}$; CBS = 1.7 and 67 nM, rG4 = 0.87 and 49 nM). These values suggest that the Sox2_HMG_ CBS and rG4 fast versus slow dissociation phases (Fig. 2C) could correspond to the complex states in the low- versus high-affinity binding curve transitions (Fig. 1B), respectively. We note for the Sox2_HMG_ G4 RNA interaction that the association was biphasic (Supplemental Fig. S3E), whereas the use of $k_{\mathrm{on}}^{\mathrm{app}}$ in these calculations corresponds to the initial association phase only.

### ERα_DBD-Ext_ multiphasic dissociation is not due to a “locked” binding conformation

We then sought a molecular model to explain the multiphasic dissociation kinetics observed for ERα_DBD-Ext_. An in vitro study of the full-length glucocorticoid receptor (GR), a nuclear hormone receptor with strong similarities to ERα, reported multiphasic dsDNA dissociation kinetics remarkably similar to the ERα_DBD-Ext_ dsDNA dissociation kinetics observed here (De Angelis et al. 2015). Those authors proposed a “locked” binding conformation model to explain GR multiphasic ligand dissociation kinetics (Supplemental Fig. S4A). This model suggests that after initial GR-dsDNA association, the complex can slowly isomerize to an alternative state but must slowly isomerize back to the initial complex state before ligand dissociation can occur. A prediction of this model is that if a brief protein–ligand incubation period is allowed before complex dissociation is induced (e.g., by competitor addition), then the complex should not have time to isomerize to the more stable alternative state, and the slow phase of the dissociation kinetics should be ablated.

To test if ERα_DBD-Ext_ ligand dissociation kinetics could be explained by the “locked” binding conformation model, we conducted FPCD experiments with variable protein–ligand incubation times. Our findings indicated that 2 versus 60 min protein–ligand incubations produced similarly biphasic ERα_DBD-Ext_ dsDNA dissociation, with slow phase contributions of 30%–40% based on biexponential regression (Supplemental Fig. S4B,C). In contrast, based on our Figure 2B data, the GR model predicts a ∼5% slow phase contribution after a 2 min incubation. The behavior was somewhat different when RNA was the ligand—the shorter incubation time did affect ERα_DBD-Ext_ hRNA dissociation, but by partially reducing the fast phase of the biexponential regression instead of the anticipated slow phase reduction (Supplemental Fig. S4D). It is notable that the ERα_DBD-Ext_ hRNA association is incomplete after a 2 min incubation at the protein concentrations used (Supplemental Fig. S3C), suggesting that the fast dissociation phase of the biexponential regression emerges during the second ERα_DBD-Ext_ hRNA association phase (Fig. 4C). Overall, despite the similarities in dissociation kinetics, our data indicate that the “locked” conformation model proposed for GR multiphasic ligand dissociation does not apply to ERα_DBD-Ext_ DNA or RNA biphasic dissociation.

**FIGURE 5.**
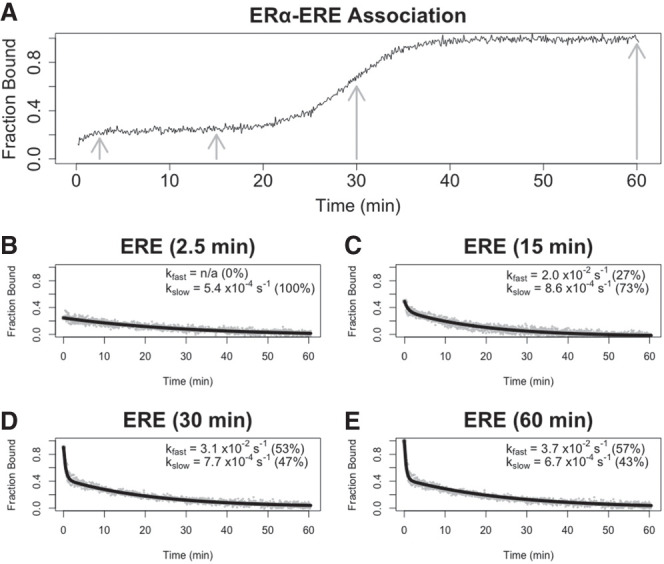
The more stable complex state forms first during multiphasic ERα_DBD-Ext_ ligand association. (*A*) Normalized ERα_DBD-Ext_ association curve. Normalized association curve of 30 nM ERα_DBD-Ext_ and 2 nM ERE dsDNA, taken from Figure 4A. Gray arrows correspond to incubation times before competitor addition in *B*–*E* experiments. (*B*–*E*) Dissociation curves from FPCD experiments in which a competitor was added after variable protein–ligand incubation times. FPCD experiments (see Fig. 2A) were performed for the ERα_DBD-Ext_–ERE interaction using 2 nM ligand, 10 µM competitor, and 30 nM protein; protein–ligand reactions were incubated for 2.5 min (*B*), 15 min (*C*), 30 min (*D*), or 60 min (*E*) before competitor addition. Anisotropy traces were normalized to the internal controls to give “Fraction Bound” over time, and then normalized data were fit with biexponential regression (Equation 5) to determine rate constants. Gray dots are data points and solid black lines are regression fits from single experiments. Percent contributions of fast versus slow components to the biexponential curve are in parentheses. Rate constant values are from single independent experiments.

Although these findings were sufficient to refute one model, we further investigated how ERα_DBD-Ext_ dsDNA complex stability varied during its multiphasic association to provide insights into alternative models. The above dsDNA experiments used ERα_DBD-Ext_ concentrations severalfold above the ligand *K*_d_, in which nucleic acid association occurs in a single apparent step (Fig. 4A). To determine if protein–ligand incubation time affects dissociation kinetics at lower protein concentrations, when the association is multiphasic, we performed FPCD experiments under these conditions using ERE dsDNA (Fig. 5). We selected protein–ligand incubation times (Fig. 5A for reference) just after initial association at the beginning of the “lag” phase (2.5 min), at the end of the “lag” phase before secondary association (15 min), toward the end of secondary association (30 min), and at binding equilibrium (60 min). These findings indicate that ERα_DBD-Ext_ ERE dsDNA dissociation is slow and monophasic during the “lag” phase after initial association, but complex dissociation acquires a faster component and becomes biphasic during the second association phase (Fig. 5B–E). Curiously, this suggests that the more stable complex state emerges first, followed by the less stable complex state, which contrasts with the positive cooperativity observed by the ERα_DBD-Ext_-ΕRΕ binding curve (Fig. 1A). Notably, this is the same trend in dissociation behavior over multiphasic association that was observed for the ERα_DBD-Ext_-XBP1 interaction above, consistent with the hypothesis that the two ligands could share an underlying mechanism.

### ERα_DBD-Ext_ multiphasic DNA dissociation is conserved across methodology and temperature

**FIGURE 6.**
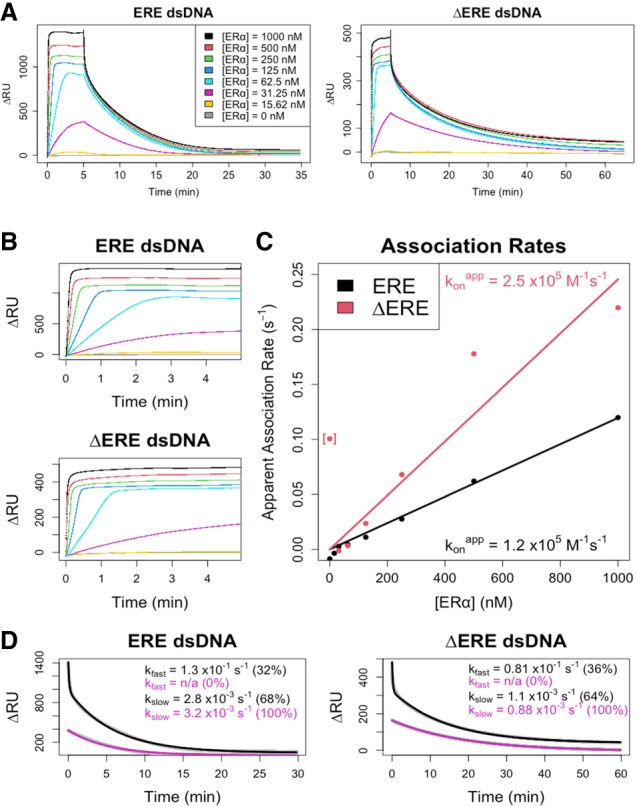
Surface plasmon resonance confirms multiphasic ERα_DBD-Ext_-dsDNA binding kinetics. (*A*) Normalized SPR traces are shown for the association and dissociation phases. The change in RU signal after ERα_DBD-Ext_ injection (ΔRU), which excludes changes observed in the empty flow cell, is shown as a function of time postinjection. Legends indicate the concentration of ERα_DBD-Ext_ used during protein injection. Lines are data, not regression fits. (*B*) ERα_DBD-Ext_ ligand association curves. SPR association phase curves, taken from *A* with the same color scheme. (*C*) Association rate analysis. Association curves from *B* had their initial slopes normalized to their signal dynamic range to calculate their apparent association rates (see Materials and Methods). Plots of apparent initial association rates versus protein concentrations were fit with zero-intercept linear regression to calculate apparent initial association rate constants ($k_{\mathrm{on}}^{\mathrm{app}}$). Dots are data and solid lines are linear regression fits. Bracketed data point is an outlier from the initial slope quantification of the 16 nM association curve in *B*, which has negligible signal dynamic range. (*D*) Dissociation rate analysis. SPR dissociation phase curves, taken from *A*, with the same color scheme. Two key protein concentrations (1 µM and 30 nM) are shown. Data were fit with biexponential regression (Equation 5) to determine rate constants. Gray dots are data and solid black/purple lines are regression fits; data points are mostly obscured by regression lines. Percent contributions of fast versus slow components to the biexponential curve are in parentheses. All SPR data in this figure are from a single experiment per ligand.

To ensure that our findings were not due to an unexpected feature of our FP experimental design, we used SPR to independently measure ERα_DBD-Ext_ dsDNA association and dissociation kinetics (Fig. 6). This also provided the opportunity to obtain data at a second temperature (25°C vs. 4°C). Given our assay requirements and the limitations of SPR, we could only measure the association kinetics up to 5 min, which is before the secondary association phase that emerged in FP assays. Using SPR association curves to calculate ERα_DBD-Ext_ apparent association rate constants for ERE and ΔERE dsDNA (Fig. 6C), we infer that they are 2× to 3× higher than the respective values determined by FP (Table 1), which puts them in good agreement given the higher temperature for SPR experiments. However, our association data have prolonged linear phases (Fig. 6B), suggesting that mass transfer effects might be deflating the apparent association rates to some unknown degree.

Based on the FP data, we predicted that SPR could be used to test the dissociation behavior at different protein concentrations. As noted above, SPR could not accommodate the time range to fully repeat the FP experiments that revealed multiphasic association (Fig. 4) or dsDNA dissociation kinetics over the time course of its multiphasic association (Fig. 5). However, we estimated that complexes formed at lower ERα_DBD-Ext_ concentrations should begin their dissociation curves in the “lag” phase of multiphasic association, whereas complexes formed at higher protein concentrations should begin their dissociation curves at equilibrium. As predicted, for both ERE and ΔERE dsDNA, the ERα_DBD-Ext_ dissociation curves were biphasic at high ERα_DBD-Ext_ concentrations but monophasic slow at low ERα_DBD-Ext_ concentrations (Fig. 6D), suggesting that our SPR and FP findings are reporting the same phenomenon. The SPR-derived rate constants for the fast and slow phases of the biexponential regressions (Fig. 6D) were 1× to 3× greater than their respective FP values (Table 1), which is in good agreement given the temperature differential. We note that the percent contribution of the fast phase to the biexponential regression was lower for SPR (32%–36%) than for FP (60%–71%). However, unlike the FP experiments (Fig. 1A), the SPR signal appeared not to have yet reached a plateau at the highest protein concentrations, and the percent contribution of the fast phase to the biexponential regression still appeared to be increasing with protein concentration (Fig. 6A), suggesting that these values may be more similar at saturating protein concentrations. Collectively, these SPR-based findings independently confirm the kinetic observations from our FP experiments.

## DISCUSSION

ERα and Sox2 have been previously demonstrated to bind RNA with structural specificity in vitro and to associate with RNA in vivo (Ng et al. 2012; Holmes et al. 2020; Xu et al. 2021; Hamilton et al. 2022, 2023). Additionally, while their RNA and DNA interactions are reportedly competitive, initial studies also suggest that the RNA- and DNA-binding surfaces do not perfectly overlap on the TFs (Holmes et al. 2020; Hou et al. 2020; Steiner et al. 2022). Our work expands on these in vitro findings by elucidating the timescales for ERα and Sox2 nucleic acid association and dissociation and by interrogating their respective RNA- versus DNA-binding mechanisms.

### A model for Sox2_HMG_ DNA and RNA binding

**FIGURE 7.**
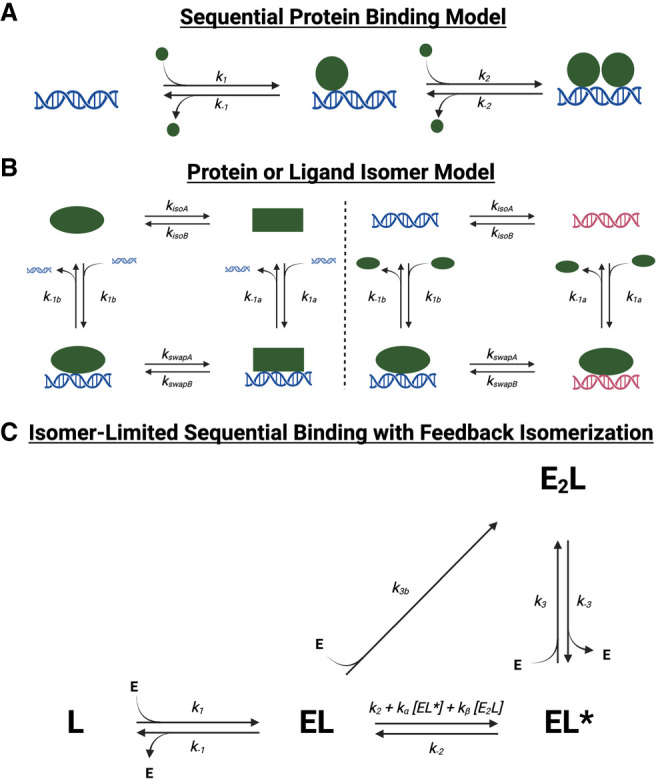
Various reaction schemes that predict biphasic protein–ligand dissociation. (*A*) Sequential protein-binding model. After initial protein–ligand association, additional protein can associate with the complex to form higher stoichiometry complexes. If the complex states with differing stoichiometries also have differing stabilities, multiphasic dissociation kinetics can be produced. If protein associates at differing rates with ligand versus existing complex, multiphasic association kinetics can be produced. Based on this model, rate constants for Sox2_HMG_ CBS dsDNA binding are inferred to be *k*_1,2_ ≈ 8 × 10^5^ M^−1^ sec^−1^, *k*_−1_ ≈ 1 × 10^−3^ sec^−1^, and *k*_−2_ ≈ 5 × 10^−2^ sec^−1^. The rate constants for Sox2_HMG_ G4 RNA binding are inferred to be *k*_1_ ≈ 1 × 10^6^ M^−1^ sec^−1^, *k*_2_ ≈ 7 × 10^4^ M^−1^ sec^−1^, *k*_−1_ ≈ 1 × 10^−3^ sec^−1^, and *k*_−2_ ≈ 5 × 10^−2^ sec^−1^. (*B*) Protein or ligand isomer model. The protein (*left*) or ligand (*right*) may isomerize to an alternative state, which produces different protein–ligand association and/or dissociation rates. (*C*) Isomer-limited sequential binding with feedback isomerization. After initial protein–ligand association to form a stable complex (*EL*), protein can inefficiently associate with the initial complex to form a higher-order stoichiometry complex (*E*_2_*L*), or the initial complex can isomerize to an alternative complex state (EL*) that can more readily accommodate additional protein monomers. Complex isomerization is intrinsically slow but may be accelerated by “feedback” from isomerized complex or higher-order stoichiometry complex. Such a reaction scheme could produce monophasic, biphasic, or “lagged” triphasic association and monophasic, biphasic, or triphasic dissociation, depending on specific values of rate constants. E is a monomer of free protein, and L is free ligand.

Our kinetic and thermodynamic data allow us to propose a minimum kinetic model for Sox2_HMG_ binding to nucleic acids. Binding to target (CBS) dsDNA and G4 RNA exhibited two-transition equilibrium binding (Fig. 1B), biphasic dissociation (Fig. 2C), and monophasic dsDNA association and biphasic G4 RNA association (Fig. 4D,E; Supplemental Fig. S3D,E). The second, lower affinity Sox2_HMG_ CBS and rG4 binding transitions have similar affinities for nontarget DNA and RNA. Prior data indicate that Sox2_HMG_ can bind DNA and RNA at protein–ligand stoichiometries higher than 1:1 (Moosa et al. 2018; Holmes et al. 2020; Hamilton et al. 2022). We propose that the simplest model to sufficiently explain these findings is a sequential protein-binding model (Fig. 7A). We also considered a ligand isomerization (but not protein isomerization) model as shown in Figure 7B that would be consistent with the data if the ligand states were in comparable proportions at equilibrium and had drastically different affinities for the protein. However, single dominant bands were observed via native-PAGE during nucleic acid preparation (see Materials and Methods), no two-transition binding curves were produced for other dsDNA ligands (Fig. 1B), and G4 RNAs are normally quite stable in vitro under our 135 mM KCl conditions (Lane et al. 2008; Crenshaw et al. 2015). Consequently, we do not favor the Figure 6B ligand isomerization model.

Under a sequential protein-binding model (Fig. 7A; see legend for inferred rate constants), Sox2_HMG_ initially binds target dsDNA or G4 RNA with high affinity, and then an additional Sox2_HMG_ monomer (or more) binds with lower affinity. Prior studies attribute this to sequence-specific (or structure-specific) binding followed by nonspecific binding (Holmes et al. 2020; Hamilton et al. 2022). We note that Sox2 monomer binding should “cover” most of the CBS dsDNA ligand with respect to linear sequence (Supplemental Fig. S1B), but this might not necessarily preclude nonspecific interactions on the opposite side of the DNA. Upon addition of competitor, the fast dissociation phase results from the less stable monomer(s) dissociating followed by slow dissociation of the more stable monomer from the ligand. Because association appears approximately monophasic for DNA and biphasic for RNA (Supplemental Fig. S3D,E), we infer that the DNA association rate constants for successive monomers are comparable, but RNA association rate constants decrease with sequential monomer association. Finally, we note that the single-transition Sox2_HMG_ binding curves for nontarget DNA and RNA support this model—without the tight binding, Sox2_HMG_ would only bind weakly with increasing *K*_d_ for each subsequent monomer. Such behavior could produce the low Hill coefficients seen for nontarget DNA and RNA (Fig. 1B), and it would explain the similar affinities for the nontarget nucleic acids’ $K_{d}^{\mathrm{app}}$ and the target DNA and RNA second transition $K_{d}^{\mathrm{app}}$.

### A model for ERα_DBD-Ext_ DNA and RNA binding

Synthesis of our thermodynamic and kinetic data allows us to consider several models for DNA and RNA binding by ERα_DBD-Ext_. ERα_DBD-Ext_ equilibrium binding exhibited a single transition with apparent positive cooperativity for ERE dsDNA but not for ΔERE dsDNA or XBP1 hRNA (Fig. 1A). Existing crystal structures of ERα_DBD_ binding to ERE dsDNA reveal that one ERα_DBD_ monomer binds each of the two repeats in the palindromic recognition sequence, and the two monomers then stabilize one another on DNA through protein–protein interactions (Schwabe et al. 1993), which explains the apparent positive cooperativity and suggests 2:1 protein–DNA stoichiometry. By extension, it seems reasonable that the loss of one recognition sequence repeat in the ΔERE dsDNA would reduce stoichiometry and cooperativity, consistent with our data (Fig. 1A). The ERα_DBD-Ext_ dsDNA and RNA interactions also exhibited biphasic dissociation with similar rate constants (Fig. 2B), and for dsDNA this persisted even in the absence of competitor (Fig. 3) or hinge residues (Supplemental Fig. S2). ERα_DBD-Ext_ dsDNA association was apparently triphasic at lower protein concentrations, with a 10–20 min “lag” between two typical association phases, whereas RNA association seemed biphasic but did not adequately fit a standard biexponential (Fig. 4A,B). Most notably, although the ERα_DBD-Ext_ dsDNA $K_{d}^{\mathrm{app}}$ was primarily predicted by the $k_{\mathrm{off}\;\mathrm{slow}}^{\mathrm{app}}$, the RNA $K_{d}^{\mathrm{app}}$ was more influenced by the $k_{\mathrm{off}\;\mathrm{fast}}^{\mathrm{app}}$.

This raises the question of what model best explains the ERα-binding data. First, a standard sequential protein-binding model for biphasic dissociation (Fig. 7A), which was the favored model for Sox2_HMG_ binding, is inconsistent with our data. Specifically, we note that this model can only explain biphasic dissociation from a state of saturated ligand binding if subsequent ERα_DBD-Ext_ monomers bind DNA/RNA much less stably than the first monomer, but ERα_DBD-Ext_ exhibits neither two-transition binding nor a low Hill coefficient (i.e., *n* < 1) (Fig. 1A). Furthermore, such a model would not be able to produce the “lagged” triphasic association curves that we observed, even if a slow isomerization step were included between sequential monomer binding events in Figure 7A (this would produce classic biphasic association). Next, the “locked” binding conformation model (Supplemental Fig. S4A), which was previously proposed to explain biphasic DNA dissociation for GR (De Angelis et al. 2015), was specifically tested and discounted by our studies (Supplemental Fig. S4). We also note that this model could explain monophasic association, or biphasic association if the “locked” complex conformation had significantly altered anisotropy, but it cannot explain our “lagged” triphasic association data (Fig. 4). Finally, the protein or ligand isomerization models shown in Figure 7B would require significantly different complex state stabilities to explain our data, but ERα_DBD-Ext_ DNA/RNA binding exhibits neither two-transition binding nor a low Hill coefficient (Fig. 1A). In addition, Figure 7B models could be reconciled with monophasic or biphasic association, but they cannot explain the “lagged” triphasic association we observed (Fig. 4). We considered if heterogeneity in ligand or protein could explain the data, but the RNA/DNA had a single dominant band via native-PAGE (see Materials and Methods) and no protein heterogeneity was observed during size-exclusion chromatography or SDS-PAGE (see Materials and Methods).

None of the above models (Fig. 7A,B; Supplemental Fig. S4A) could explain the “lagged” triphasic association we observed (Fig. 4). In contrast, one kinetic phenomenon that can produce an apparent association lag followed by seemingly rapid/spontaneous association involves sequential reactions in which a downstream product has a “feedback” effect to catalyze an earlier step in the reaction. In the case of ERα_DBD-Ext_ dsDNA binding, such a minimum kinetic model might resemble that shown in Figure 7C. In this model, ERα_DBD-Ext_ initially binds dsDNA with high affinity to produce a stable complex. We note that it is possible for the initial complex to have 2:1 instead of the 1:1 protein–ligand stoichiometry shown without drastically altering apparent kinetics. Additional ERα_DBD-Ext_ monomers would be capable of inefficiently associating with the initial stable complex, but the initial complex could also slowly isomerize to an alternate complex state that better accommodates additional ERα_DBD-Ext_ monomer binding. Critically, this complex isomerization would have to be susceptible to acceleration by an already isomerized (and/or higher-order stoichiometry) complex. Our simulations with the Figure 7C model suggest that it can recapitulate the kinetic trends observed for ERα_DBD-Ext_ dsDNA binding (Supplemental Fig. S5, see legend). Notably, accelerating the reverse isomerization (i.e., increasing *k*_−2_) in simulations made the data look more like the ERα_DBD-Ext_ RNA binding kinetics (Supplemental Fig. S6, see legend). In essence, our observations that ERα_DBD-Ext_ DNA and RNA binding had similar dissociation rates and initial association rates despite differing $K_{d}^{\mathrm{app}}$, Hill coefficients, and association curve shapes could be recapitulated by this model by changing a single rate constant value.

Overall, we present evidence that ERα_DBD-Ext_ DNA and RNA binding cannot be adequately explained by traditional reaction schemes. Instead, we provide a “framework” model that can generally recapitulate ERα_DBD-Ext_ DNA and RNA kinetic trends. We note that our few simulations with this model are certainly not a perfect fit to the experimental data herein. Indeed, the “flexibility” of the model made it difficult to exhaustively fit our experimental data via iterative numerical integration and regression. Thus, it is likely that our Figure 7C model does not completely capture the mechanism(s) of ERα_DBD-Ext_ DNA and RNA binding. Rather, the model represents a starting point for insights into the “true” mechanism. First, the model suggests that, despite the seemingly disparate ERα_DBD-Ext_ DNA versus RNA association behaviors and affinities, they could share a reaction mechanism. Second, the hallmark “lagged” triphasic association we observed is critically dependent on the reaction mechanism “feedback” and complex isomerization in our simulations. Although it is not hard to imagine a TF like ERα that binds gene targets as a homodimer having more than one conformational state after target binding, the novel implication that some ERα_DBD-Ext_ nucleic acid–binding states can influence the stability of other complex states warrants further investigation. Such a mechanism could be especially relevant in situations like ERα nucleic acid condensates where multiple complexes are crowded together (Nair et al. 2019), because overall condensate stability and architecture could be impacted if certain complex states influence the stabilities of other complex states and their ability to accommodate additional protein monomers.

### Concluding remarks

In a cell, hundreds to thousands of TFs search for their unique target DNA sequences to perform critical regulation of gene expression. During this target search, the TFs not only coordinate with many protein-binding partners, but they are also inundated with numerous other nucleic acids like the nontarget DNA in surrounding chromatin and free and nascent RNA. Consequently, TFs likely experience a variety of transitory nucleic acid–binding events on the way to their target DNA sites. This is likely to be even more pronounced in the dense environment of biological condensates. Discerning the biological relevance of a TF's numerous nucleic acid interactions requires careful consideration of the prevalence, lifetimes, reaction mechanism(s), and interligand influences of these varied binding events. Our biophysical studies herein estimate the timescales of association and dissociation events for the RNA and DNA interactions of two model TFs, and they also provide insight into the reaction mechanism(s) for these TF–nucleic acid interactions. Thus, our findings represent a valuable “touchstone” for considerations of how the target site searches of ERα, Sox2, and other related TFs are influenced by competing nucleic acids like RNA.

## MATERIALS AND METHODS

### Protein expression and purification

For recombinant Sox2_HMG_, a plasmid encoding Sox2_HMG_ as an N-terminal octa-histidine and maltose-binding protein (MBP) fusion with rhinovirus 3C protease cleavage site in a pET30b vector was generously provided by Desmond Hamilton (Batey laboratory, University of Colorado Boulder) (Hamilton et al. 2022). The plasmid was transformed into BL21(DE3) *Escherichia coli* bacterial cells, inoculated into 20 mL media (LB broth with 100 µg/mL kanamycin), and then the starter culture was incubated overnight at 37°C/200 rpm until *A*_600_ ≈ 5.0. Starter culture was diluted to *A*_600_ ≈ 0.1 in 1 L media, and then incubated at 37°C/200 rpm for ∼2 h until *A*_600_ ≈ 0.5–0.8. The culture was then induced with 0.5 mM isopropyl β-d-thiogalactopyranoside (IPTG) and incubated at 37°C/200 rpm for an additional 4 h. Following induction, the culture was pelleted by centrifugation (4000*g*/4°C/20 min) and resuspended in 50 mL Amylose A buffer (20 mM Tris pH 7.5 at 25°C, 200 mM NaCl, 1 mM EDTA) with one Pierce Protease Inhibitor Tablet (Thermo Scientific A32965) and 50 mg lysozyme (Sigma-Aldrich L6876), and then lysed with an Emulsiflex C3 homogenizer (Avestin) at 15,000–18,000 psi. Lysate was clarified by centrifugation (27,000*g*/4°C/30 min) and the supernatant was collected. A 4°C AKTA Pure FPLC system (Cytiva) was prepared with a 10 mL amylose column (NEB E8021S) and 2 mL/min flow rate, equilibrated with Amylose A buffer, supernatant applied, washed with Amylose A buffer, and eluted with Amylose B buffer (20 mM Tris pH 7.5 at 25°C, 200 mM NaCl, 1 mM EDTA, 10 mM maltose). To the eluent was added 1.0 mg of Prescission Protease, and it was then loaded into 10 kDa-cutoff SnakeSkin Dialysis Tubing (Thermo Scientific 68100) and dialyzed overnight at 4°C in P-cell A buffer (50 mM Tris pH 7.5 at 25°C, 1 mM EDTA) with 50 mM NaCl. The FPLC system was prepared with a 10 mL P11-phosphocellulose column (Whatman) and 2 mL/min flow rate, equilibrated with P-cell A buffer, dialyzed eluent applied, washed with P-cell A buffer, and eluted with a 100 mL 0%–100% P-cell B buffer (50 mM Tris pH 7.5 at 25°C, 1 mM EDTA, 1 M NaCl) gradient. Protein-containing (via *A*_280_) fractions were reconciled and concentrated with a 10 kDa-cutoff centrifugal filter unit. The FPLC system was prepared with a Superose 6 size-exclusion column and 0.25 mL/min flow rate, and then equilibrated with Sizing Buffer (10 mM Tris pH 7.5 at 25°C, 250 mM NaCl, 1 mM EDTA), concentrated eluent applied, and followed with Sizing Buffer. Protein-containing (via *A*_280_) eluent fractions were reconciled for the final product, and then flash-frozen in liquid nitrogen and stored at −80°C. SDS-PAGE indicated ≥95% purity, and protomer concentrations were determined by spectroscopy with *ε*_280_ = 13,980 M^−1^ cm^−1^. One liter of culture typically yielded ∼4 mg of final protein.

For recombinant ERα_DBD_ and ERα_DBD-Ext_ (residues 180–262 for ERα_DBD_, 180–280 for ERα_DBD-Ext_), proteins were expressed with a thrombin-cleavable N-terminal hexahistidine tag using a pET28a (EMD Biosciences) vector. Protein expression and purification methods were described previously (Steiner et al. 2022). Starting with a single transformed colony of BL21(DE3)pLysS *E. coli*, expression cultures were grown at 37°C (with 50 μg/mL kanamycin and 50 μg/mL chloramphenicol) using 2× YT rich media to an OD_600_ of 0.8–1.0, and cold shocked on ice for 20 min. IPTG was added to a final concentration of 0.4 mM, along with 50 μM ZnCl_2_, to induce protein expression, and cultures were grown for 3 h at 37°C in a shaker. Cells were harvested by centrifugation (5000*g*) and pellets were stored at −20°C. Cell pellets were thawed and resuspended in 50 mL lysis buffer (20 mM Tris pH 7.5 at 25°C, 1 M NaCl, 10 mM imidazole pH 7.5, 5% glycerol) per 1 L of cells with one EDTA-free protease inhibitor cocktail tablet (Roche). Cells were lysed using a Misonix Sonicator 3000 (110 W for 2 min total ON-time, pulse: 15 sec ON/45 sec OFF, ½ in tip) and the lysate cleared by centrifugation (15,000*g*, 30 min). Cleared lysate was loaded onto lysis buffer–equilibrated Ni-NTA resin (GoldBio, 5 mL resin per 50 mL lysate) and rocked gently for 1 h at 4°C. The bead slurry was loaded onto a gravity flow column and washed twice with increasing concentrations of imidazole in lysis buffer (wash 1: 20 mM imidazole, wash 2: 30 mM imidazole), and then eluted with 300 mM imidazole in lysis buffer. Bovine α-Thrombin (Haematologic Technologies) was added (10 U/mg protein) to the eluate to remove the hexahistidine tag. The eluate solution was transferred to 6–8 kDa MWCO dialysis tubing (Spectra/Por, Spectrum Labs) and dialyzed overnight at 4°C in 4 L of column buffer (20 mM Tris pH 7.5 at 25°C, 100 mM NaCl, 5% glycerol, 1 mM DTT). Dialyzed eluate was filtered to 0.2 μm and concentrated using 5 kDa MWCO spin concentrators (Vivaspin Turbo). The sample was again filtered to 0.2 µm and loaded onto a HiLoad 16/600 Superdex 75 column (GE HealthCare) and eluted as a monomer. Pooled fractions containing recombinant ERα were assessed for purity, aliquoted, flash-frozen, and stored at −70°C. One liter of culture typically yielded 2 mg of purified protein as measured by absorption (*ε*_280_ = 14,440 M^−1^ cm^−1^). All experiments used ERα_DBD-Ext_ protein, unless otherwise indicated.

### Preparation of oligonucleotides

All oligonucleotides except the XBP1 hRNA were ordered from IDT (Coralville, IA), and their sequences in IDT syntax are provided (Supplemental Table S1). For Sox2_HMG_ dsDNA ligands, complementary oligonucleotides ordered from IDT were mixed at 100 µM each in annealing buffer (50 mM Tris pH 7.5 at 25°C, 200 mM NaCl) and subjected to a thermocycler program (95°C for 10 min, 95 → 4°C at 0.5°C/min, hold at 4°C) for annealing. For ERα dsDNA, the complementary strands were combined at 1 μM labeled and 5 μM unlabeled (ligand prep) or 100 µM each unlabeled (competitor prep) in annealing buffer (20 mM Tris pH 7.5 at 25°C, 50 mM NaCl), and then annealed by benchtop slow cooling (heated 95°C for 1 min, and then cooled to room temperature for 3 h). Complete annealing for all oligonucleotides was confirmed via native-PAGE. Concentrations of all ligands were confirmed spectroscopically using manufacturer-provided extinction coefficients.

According to prior methodology (Steiner et al. 2022), XBP1 hRNA was prepared by in vitro transcription (IVT) with T7 RNA polymerase, using dsDNA templates containing a T7 polymerase promoter sequence, which were created via PCR with IDT-synthesized oligonucleotides. Briefly, full PCR amplification was confirmed on 2% agarose gel, and subsequent IVTs were performed for 3 h at 37°C. Successful transcription was confirmed via 10%–18% denaturing PAGE. After IVT, RNAs were precipitated in 1/10th volume 3 M sodium acetate and 2.5 volumes ice cold ethanol overnight. The following day ethanol-precipitated RNA was pelleted and dried, and then resuspended and purified by denaturing urea-polyacrylamide gel electrophoresis followed by buffer exchange and concentration in a 5 kDa MWCO spin concentrator (Vivaspin Turbo). Purified RNA oligonucleotides were 3′-end labeled with pCp-AF488 (Alexa Fluor). Two hundred picomoles of RNA and 2400 pmol fluorophore were combined in labeling buffer (1 mM ATP, 10% DMSO, 50% PEG, 40 U T4 ligase and 1× T4 ligase buffer) overnight at 16°C. The labeled RNA was purified using RNA Clean & Concentrator kit (Zymo 4060), passed through a G-25 spin column, and stored at −20°C. Concentration was determined by *A*_260_ and total RNA yield was typically 10%–50% with ∼70% labeling efficiency. Purity of the final sample was assessed by 10%–15% denaturing PAGE and imaged by fluorescence (Ex = blue wavelength filter, Em = green wavelength filter). RNA samples were prepared for binding assays by fast refolding at 1 μM by snap-cooling (95°C for 1 min, ice for >5 min).

### FP-based $K_{d}^{\mathrm{app}}$ and association rate determination

Prereaction mix was prepared with 5 nM ligand in ERα-binding buffer (20 mM Tris pH 7.5 at 25°C, 100 mM NaCl, 5% glycerol, 0.01% IGEPAL) or Sox2-binding buffer (10 mM Tris pH 7.5 at 25°C, 135 mM KCl, 15 mM NaCl, 0.1 mg/mL nonacetylated BSA, 4% Ficoll, 0.05% NP-40, 1 mM DTT), and then dispensed in 36 μL volumes into the wells of a 384-well black microplate (Corning 3575). A range of protein concentrations was prepared at 10× the final reaction concentrations via serial dilution in respective binding buffer. Protein and microplates were then thermally equilibrated at 4°C for 30 min. After thermal equilibration, reactions were initiated by the addition of 4 μL of the respective 10× protein concentration to the corresponding microplate well, and then incubated for ≥60 min at 4°C. Fluorescence anisotropy readings were taken over the course of incubation immediately after protein addition (in <10 sec intervals) with a TECAN Spark microplate reader (Ex = 481 ± 20 nm, Em = 526 ± 20 nm). Each experiment had one reaction (well) per protein concentration, and one to three independent experiments were performed per protein–polynucleotide interaction.

For equilibrium dissociation constant calculations, the last 10 min of data points from the anisotropy versus time data of each reaction were averaged to give equilibrium values, and then equilibrium anisotropy values versus protein concentration data were regressed with Equation 2 (Sox2_HMG_-CBS and Sox2_HMG_-rG4) or Equation 1 (all other interactions) to determine $K_{d}^{\mathrm{app}}$ and *n*. For the displayed Sox2_HMG_-binding curves (Fig. 1B), traces were normalized to the respective maximum and minimum signals determined by regression. Mean and error (50% range) are reported in Table 1.

For initial association rate constant calculations, anisotropy versus time data for each protein concentration were fit with a smoothing spline and the initial slope of the regression was divided by the dynamic range in anisotropy for the regression to calculate apparent association rates. Apparent association rate versus protein concentration data were pruned to include only the initial linear phases and to exclude the higher protein concentrations with incomplete curves. Then, pruned data were regressed with zero-intercept linear regression to determine $k_{\mathrm{on}}^{\mathrm{app}}$. Mean and error (50% range) are reported in Table 1.

For the monophasic or biphasic association fits in Supplemental Figure S3, anisotropy versus time data for each protein concentration were pruned to only include the initial data points shown in respective graphs in Supplemental Figure S3, and then pruned data were regressed with Equation 4 (ERα_DBD-Ext_-XBP1, Sox2_HMG_-rG4) or Equation 3 (all others). Analyses were performed in R v4.3.1.

### FP-based competitive dissociation

Prereaction mix was prepared with 5 nM ligand in ERα (20 mM Tris pH 7.5 at 25°C, 100 mM NaCl, 5% glycerol, 0.01% IGEPAL) or Sox2 (10 mM Tris pH 7.5 at 25°C, 135 mM KCl, 15 mM NaCl, 0.1 mg/mL nonacetylated BSA, 4% Ficoll, 0.05% NP-40, 1 mM DTT) binding buffer, and then dispensed in 32 μL volumes into the wells of a 384-well black microplate (Corning 3575). Protein was prepared at 10× the reaction concentrations of 30 nM (ERα_DBD-Ext_-dsDNA; Fig. 5), 100 nM (ERα_DBD-Ext_-dsDNA; Fig. 2), or 500 nM (ERα_DBD-Ext_-RNA and Sox2_HMG_). Competitor was prepared at 10× the reaction concentration of 10 µM (i.e., 100 µM). Microplates (ligand), protein, and competitor were then thermally equilibrated at 4°C for 30–60 min. After thermal equilibration, 4 µL of 10× protein or binding buffer (baseline control) was added to the microplate wells, and then the reactions were incubated at 4°C for 1 h or indicated (Figs. 4 and 5) shorter times. After protein–ligand incubation, 4 µL of 10× competitor or binding buffer (max signal control) was added to the microplate wells, and then incubated for ≥60 min at 4°C. Fluorescence anisotropy readings were taken over the course of incubation immediately after protein addition (in <10 sec intervals) with a TECAN Spark microplate reader (Ex = 481 ± 20 nm, Em = 526 ± 20 nm). Figure 2A provides helpful clarification of the methodology. Two internal controls were used: control-1, which used buffer controls for protein addition in step-1 (Fig. 2A) and competitor addition in step-2 (Fig. 2A), and control-2, which used a buffer control for competitor addition in step-2 (Fig. 2A). Each experiment had two to three reaction replicates per condition/control, and two independent experiments were performed per protein–polynucleotide interaction.

Anisotropy versus time data for the experimental reaction was normalized to the data for the two internal controls to give fraction bound versus time. For reactions with shorter (<1 h) protein–ligand incubation times, an additional internal max signal control was always included in which protein–ligand incubation was ≥1 h, and this was used for normalization to calculate fraction bound. Fraction bound versus time data were regressed with Equation 5 to determine *k*_fast_, *k*_slow_, and β_fast_. For regression, the initial fraction bound (*A*_max_) was constrained to the initial value calculated for the internal control with identical protein–ligand incubation that omitted competitor addition. In cases in which dissociation was approximately slow monophasic, the regression was additionally constrained such that *k*_fast_ = 0 and β_fast_ = 0. Analyses were performed in R v4.3.1. Across experiments, percent contributions of the biexponential typically varied <15%.

### FP-based jump dilution

Prereaction mix was prepared with 50 nM ligand in binding buffer (20 mM Tris pH 7.5 at 25°C, 100 mM NaCl, 5% glycerol, 0.01% IGEPAL), and with (experimental reaction) or without (baseline control) 50 nM protein. Wells of a 384-well black microplate (Corning 3575) were filled with 79 µL binding buffer (experimental reaction and baseline control) or 50 nM protein (max signal control), and then the microplates and prereaction mixes were incubated at 4°C for 1 h. After incubation, 1 µL of protein–ligand mix was diluted in the buffer-only (experimental reaction) or 50 nM protein (max signal control) microplate wells, and ligand-only mix was diluted in buffer-only microplate wells (baseline control), and then the reactions incubated at 4°C for 1 h. Fluorescence anisotropy readings were taken over the course of incubation immediately after dilutions (in <10 sec intervals) with a TECAN Spark microplate reader (Ex = 481 ± 20 nm, Em = 526 ± 20 nm). Two controls are used: control-1, which uses a buffer control for protein addition, and control-2, which uses an equimolar protein control for buffer dilution. Each experiment had one reaction (well) per condition/control, and three independent experiments were performed.

Anisotropy versus time data for the experimental reaction were normalized to the data for the two internal controls to give fraction bound versus time. Fraction bound versus time data were regressed with Equation 5 to determine *k*_fast_, *k*_slow_, and β_fast_. For regression, the initial fraction bound (*A*_max_) was constrained to a value of 1. Analyses were performed in R v4.3.1. Across experiments, percent contributions of the biexponential typically varied <15%.

### Surface plasmon resonance

Streptavidin-coated S-series chips were purchased commercially (Xantec SCBS-SAD200M) and docked into a Biacore T200 SPR instrument (Cytiva). Before first-time use, all four chip flow cells (FCs) were washed (25 µL/min for 1 min) five times with Activation Buffer (50 mM NaOH, 1 M NaCl) to remove any unbound streptavidin, and then washed (25 µL/min for 10 min) with Running Buffer (20 mM Tris pH 7.5 at 25°C, 100 mM NaCl, 5% glycerol, 0.01% IGEPAL) to ensure surface stability. Ligand immobilization of biotin-labeled ERE (FC-2) or ΔERE (FC-4) dsDNA was performed by flowing 20 nM ligand solutions over the respective FC at 1 µL/min until a ΔRU of 300 was achieved, and then priming the system with Running Buffer three times. FCs 1 and 3 were used as background controls for FC 2 and 4 experiments, respectively.

For kinetic experiments, the indicated (Fig. 6A) ERα_DBD-Ext_ concentrations were flowed sequentially over the control and ligand FCs at 70 µL/min for 5 min to monitor association, followed by a 30–60 min wash phase (70 µL/min) with Running Buffer to monitor dissociation. ERα_DBD-Ext_ concentrations were tested in increasing order, and the FCs were washed (70 µL/min for 1 min) between ERα_DBD-Ext_ concentrations one time with Regeneration Buffer (1 M NaCl) and three times with Running Buffer. Control FC signal was subtracted from the experimental FC signal to generate adjusted signal versus time data, and then adjusted data were exported from the instrument.

Adjusted data for each protein concentration were subjected to baseline subtraction to generate ΔRU versus time data. For initial association rate constant calculations, ΔRU versus time data were pruned to include only the association phases, and then time points were adjusted to start at zero time. For each protein concentration, pruned association data were used to calculate the change in ΔRU over the first 3 sec of association, and then this ΔΔRU was normalized to the dynamic range in ΔRU for each protein concentration to calculate apparent association rates. Apparent association rate versus protein concentration data were regressed with zero-intercept linear regression to determine $k_{\mathrm{on}}^{\mathrm{app}}$. For dissociation rate calculations, ΔRU versus time data were pruned to include only the dissociation phases, and then time points were adjusted to start at zero time. Pruned dissociation data for each protein concentration were regressed with Equation 5 to determine *k*_fast_, *k*_slow_, and β_fast_. For regression, the initial signal (*A*_max_) was constrained to the final ΔRU observed at the end of the preceding SPR association phase. For cases in which dissociation was approximately slow monophasic, regression was additionally constrained such that *k*_fast_ = 0 and β_fast_ = 0. Analyses were performed in R v4.3.1.

### ERα reaction scheme simulations

Reactions (Supplemental Figs. S5 and S6) were simulated and analyzed in R v4.3.1 using deSolve::ode (package::function) and the lsoda integrator (Soetaert et al. 2010). Numerical integration of the Equations 6–10 system of differential equations was performed with a given integration time step (Δ*t*) in two phases with fixed rate constant values from Figure 5C. In phase 1 (the association phase), for initial conditions the total protein ([*E*_T_]) and ligand ([*L*_T_]) were included as free protein and ligand and all other reactant concentrations were set to zero, and then the reactions were simulated for a given association time (*t*_on_). In phase 2, the initial conditions were set to the final reactant concentrations from phase 1 divided by a given dilution factor (*d*_f_), and then the reactions were simulated for a given dissociation time (*t*_off_). Next, relative predicted anisotropy over time was calculated from reactant concentrations over time via Equation 11. Equilibrium values were taken from end points in phase 1 simulations. Supplemental Figure S5 simulations used Δ*t* = 25 msec, *t*_on_ = 1.5 h, *t*_off_ = 1 h, *d*_*f*_ = 10^6^, [*E*_T_] = 2^−12:0^ µM, [*L*_T_] = 5 nM, *k*_1_ = 10^6^ M^−1^ sec^−1^, *k*_−1_ = 1.6 × 10^−3^ sec^−1^, *k*_2_ = 10^−5^ M^−1^ sec^−1^, *k*_−2_ = 3.2 × 10^−3^ sec^−1^, *k*_3_ = 10^9^ M^−1^ sec^−1^, *k*_−3_ = 2 × 10^−2^ sec^−1^, *k*_3*b*_ = 10^4^ M^−1^ sec^−1^, *k*_α_ = 2 × 10^7^ M^−1^ sec^−1^, *k*_β_ = 2 × 10^6^ M^−1^ sec^−1^. Supplemental Figure S6 simulations were identical, except *k*_−2_ = 10 sec^−1^.

### Equations

For Equation 1, *A* is the signal (anisotropy, ΔRU, fraction bound, etc.), *A*_min_ is the minimum signal, *A*_max_ is the maximum signal, *E*_T_ is the total protein concentration, *K*_d_ is the (apparent) equilibrium dissociation constant, and *n* is the Hill coefficient, and for Equation 2, *L*_T_ is the total ligand concentration, *K*_d1_ is the equilibrium dissociation constant of the first binding state, *K*_d2_ is the equilibrium dissociation constant of the second binding state, α is the proportion of the signal dynamic range attributable to the first binding state, and the remaining parameters are as defined for Equation 1:

(1) $$A=A_{\min}+(A_{\max}-A_{\min})\frac{E_{T^{n}}}{E_{T^{n}}+K_{d^{n}}},$$

(2) $$A=A_{\min}+(A_{\max}-A_{\min})\left(\alpha \frac{E_{T}+L_{T}+K_{d1}-\sqrt{{\left(E_{T}+L_{T}+K_{d1}\right)}^{2}-4\;E_{T}\;L_{T}}}{2\;L_{T}}+(1-\alpha )\frac{E_{T}}{E_{T}+K_{d2}}\right).$$

For Equation 3, *A*_*t*_ is the signal (anisotropy, ΔRU, fraction bound, etc.) at a given time (*t*), *A*_min_ is the minimum signal, *A*_max_ is the maximum signal, *k*_on_ is the rate constant for the association curve, and *t* is the time, and for Equation 4, *k*_fast_ is the rate constant for the fast phase of the association curve, *k*_slow_ is the rate constant for the slow phase of the association curve, β_fast_ is the proportion of the signal dynamic range attributable to the fast phase of the association curve, and remaining parameters are as defined for Equation 3:

(3) $$A_{t}=A_{\min}+(A_{\max}-A_{\min})(1-\;e^{-k_{\mathrm{on}}t}),$$

(4) $$A_{t}=A_{\min}+(A_{\max}-A_{\min})(1-{\text{β}}_{\mathrm{fast}}\;e^{-k_{\mathrm{fast}}t}-(1-{\text{β}}_{\mathrm{fast}})\;e^{-k_{\mathrm{slow}}t}).$$

For Equation 5, *A*_*t*_ is the signal (anisotropy, ΔRU, fraction bound, etc.) at a given time (*t*), *A*_min_ is the minimum signal, *A*_max_ is the maximum signal, *t* is the time, *k*_fast_ is the rate constant for the fast phase of the dissociation curve, *k*_slow_ is the rate constant for the slow phase of the dissociation curve, and β_fast_ is the proportion of the signal dynamic range attributable to the fast phase of the dissociation curve:

(5) $$A_{t}=A_{\min}+(A_{\max}-A_{\min})({\text{β}}_{\mathrm{fast}}\;e^{-k_{\mathrm{fast}}t}+(1-{\text{β}}_{\mathrm{fast}})\;e^{-k_{\mathrm{slow}}t})$$

For Equations 6–11, rate constants are defined in Figure 7C, *E* is the protein, *L* is the ligand, conjugations of these reactants are complexes, equations give rates of change for indicated reactants as a function of time (*t*), bracketed terms indicate concentrations, and *A*_rel_ is the relative predicted anisotropy:

(6) $${\left[E\right]}_{t}^{\prime }=k_{-1}{\left[EL\right]}_{t}+k_{-3}{\left[E_{2}L\right]}_{t}-{\left[E\right]}_{t}(k_{1}{\left[L\right]}_{t}+k_{3}{\left[EL*\right]}_{t}),$$

(7) $${{\left[L\right]}^{\prime }}_{t}=k_{-1}{\left[EL\right]}_{t}-k_{1}{\left[E\right]}_{t}{\left[L\right]}_{t},$$

(8) $${{\left[EL\right]}^{\prime }}_{t}=k_{1}{\left[E\right]}_{t}{\left[L\right]}_{t}+k_{-2}{\left[EL*\right]}_{t}-{\left[EL\right]}_{t}(k_{-1}+k_{2}+k_{\alpha }{\left[EL*\right]}_{t}+k_{\text{β}}{\left[E_{2}L\right]}_{t}+k_{3b}{\left[E\right]}_{t}),$$

(9) $${{\left[EL*\right]}^{\prime }}_{t}=(k_{2}+k_{\alpha }{\left[EL*\right]}_{t}+k_{\text{β}}{\left[E_{2}L\right]}_{t}){\left[EL\right]}_{t}+k_{-3}{\left[E_{2}L\right]}_{t}-{\left[EL*\right]}_{t}(k_{-2}+k_{3}{\left[E\right]}_{t}),$$

(10) $${{\left[E_{2}L\right]}^{\prime }}_{t}=k_{3}{\left[E\right]}_{t}{\left[EL*\right]}_{t}+k_{3b}{\left[E\right]}_{t}{\left[EL\right]}_{t}-k_{-3}{\left[E_{2}L\right]}_{t},$$

(11) $$A_{\mathrm{rel}}=\frac{[E_{2}L]+\frac{1}{2}(\left[EL*]+[EL\right])}{\left[E_{2}L]+[EL*]+[EL]+[L\right]}.$$

### Diagram, reaction scheme, and figure generation

All diagrams and reaction schemes were prepared on BioRender.com, tables were prepared with Word (Microsoft), graphs were prepared with R v4.3.1, RNA secondary structure predictions were done with mfold (Zuker 2003), and figures were assembled in PowerPoint (Microsoft).

## DATA DEPOSITION

The bacterial expression plasmid for Sox2_HMG_ is available from the laboratory of Robert Batey (University of Colorado Boulder, Department of Biochemistry). Our bacterial expression plasmids for ERα_DBD(-Ext)_ are available upon request (contact D.S.W.). Our R script for simulating Figure 7C reactions is available on GitHub (github.com/whemphil/ER-Sox2_Manuscript).

## SUPPLEMENTAL MATERIAL

Supplemental material is available for this article.

## COMPETING INTEREST STATEMENT

T.R.C. declares consulting status for Storm Therapeutics, Eikon Therapeutics, and SomaLogic. The other authors have no competing interests to declare.

## Figures and Tables

**Jackson R. Kominsky:**
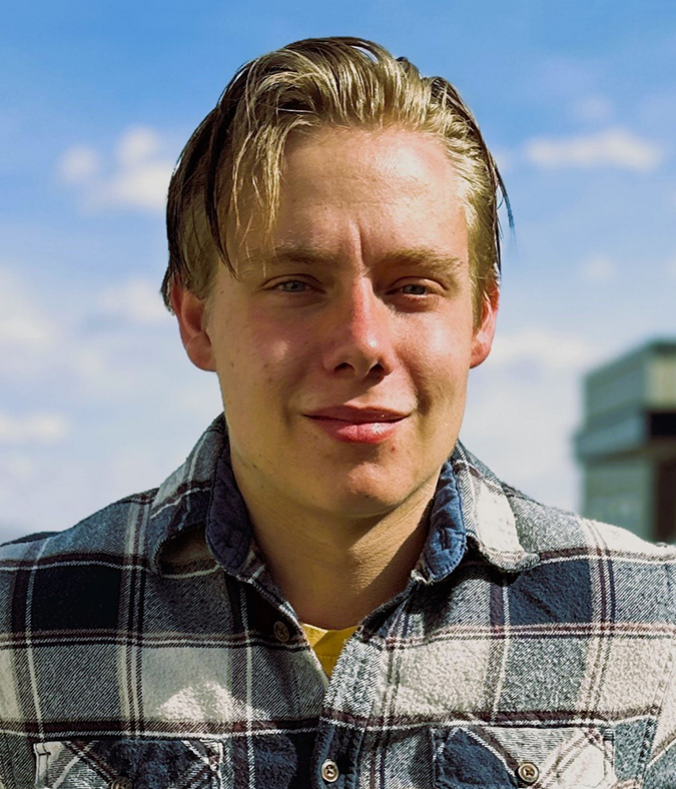


**Wayne O. Hemphill:**
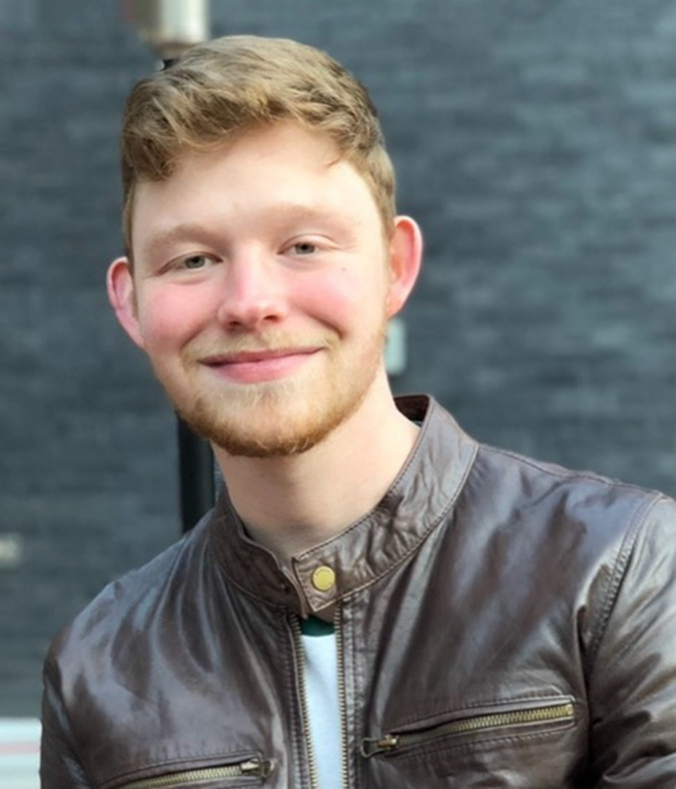


**Halley R. Steiner:**
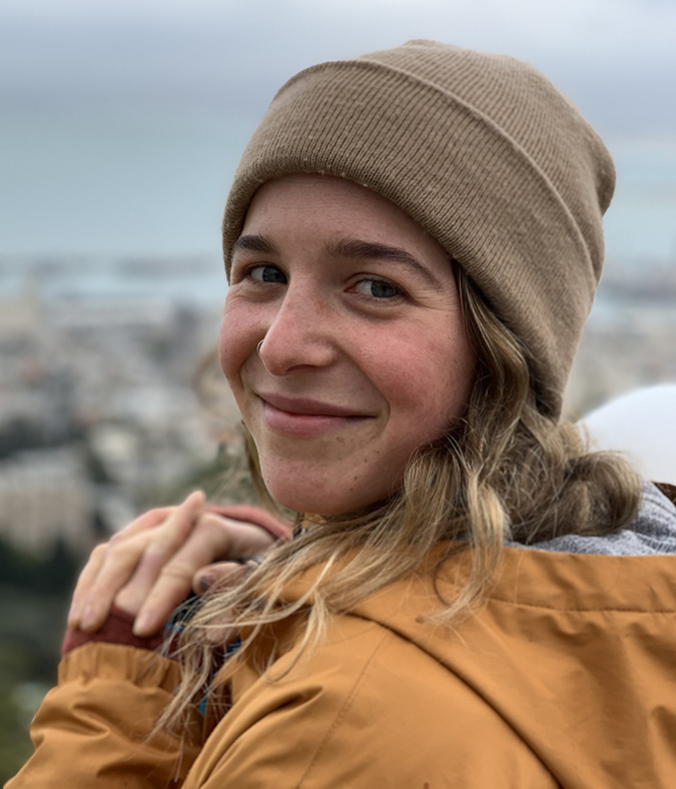


## MEET THE FIRST AUTHORS

***Meet the First Author(s)* is an editorial feature within *RNA*, in which the first author(s) of research-based papers in each issue have the opportunity to introduce themselves and their work to readers of *RNA* and the RNA research community. Wayne O. Hemphill, Halley R. Steiner, and Jackson R. Kominsky are co-first authors of this paper, “Transcription factors ERα and Sox2 have differing multiphasic DNA- and RNA-binding mechanisms.” Wayne O. Hemphill is a postdoc in Thomas Cech's laboratory at CU Boulder. His research focuses on the biophysical mechanisms of protein–nucleic acid interactions. Halley Steiner is a graduate student in Deborah Wuttke's laboratory in the Biochemistry department at CU Boulder. Her research focuses on the role of RNA binding in regulating the transcription factor estrogen receptor α. Jackson Kominsky is an undergraduate student in the Cech laboratory at the University of Colorado Boulder. His research focuses on the transcription factor Sox2 and its interaction with both DNA and RNA.**

**What are the major results described in your paper and how do they impact this branch of the field?**

Our results indicate that two transcription factors, ERα and Sox2, exhibit multiphasic RNA- and DNA-binding kinetics. For Sox2, RNA and DNA binding is kinetically similar, and appears to involve sequential monomer association. For ERα, a minimal reaction model was not clear, but our simulations suggest that it may have multiple RNA- and DNA-bound states that could influence one another's stabilities. For both transcription factors, our work represents an estimation of the lifetimes of these different complexes and their relative stabilities, which is important in thinking about the regulatory role(s) of RNA binding. For ERα specifically, our work sheds light on a previously unappreciated level of complexity in the DNA- and RNA-binding mechanisms, and it provides initial hypotheses for further research.

**What led you to study RNA or this aspect of RNA science?**

**WOH:** I love thinking about the details of how biomolecules interact with one another to create the complex networks and macroscopic effects we can observe. RNA is an especially interesting participant in such interactions, since it can be a messenger, a scaffold, or a sponge, can catalyze reactions, and more! It's an interesting variable to have in your research, and I think that's what I find attractive about studying it.

**HRS:** RNA has always been the focus of my research—I can't seem to stay away! I first studied mRNA turnover in yeast, then double-stranded RNA viral replication, RNP condensate assembly, and now the role of RNA binding to transcription factors. I've developed a deep appreciation for the complexity and breadth of roles RNA plays and enjoy how the field must constantly expand and adapt to findings that challenge our framework of basic biology.

**JRK:** One of my favorite aspects of research in the biological sciences is learning about the complex interplay between different systems and molecules, and how these systems all work concurrently. To this end, RNA has shown itself to be an incredibly versatile molecule, suggested to play a part in many different processes. I think it is an incredibly rewarding process to try and contribute novel information to a field evolving as quickly as this one.

**During the course of these experiments, were there any surprising results or particular difficulties that altered your thinking and subsequent focus?**

**WOH:** I'd say this publication was full of surprises for me, especially with respect to ERα. How do you get that weird, lagged association pattern? I'd only seen those kinds of kinetics in prion research or those old oscillating reaction chemistry demonstrations—I thought it was an artifact the first time it popped up with a transcription factor! Why did the RNA and DNA ligands behave so similarly in most regards, with comparable on and off rates, but generate such drastically different binding affinities? That one is still fun to think about. I'll certainly be even more wary in the future when interpreting apparent binding affinities!

**HRS:** When we first began this study, we were investigating if transcription factors engage in the phenomenon of direct transfer, wherein a protein exchanges nucleic acid ligands by simultaneously binding both species via an unstable ternary complex. Instead of detecting direct transfer, we observed the biphasic dissociation phenomenon. This left us completely perplexed for a good period of time, as we tested model after model. However, the effort ultimately led to uncovering a novel mechanism of transcription factor–nucleic acid exchange.

**What are some of the landmark moments that provoked your interest in science or your development as a scientist?**

**JRK:** I think I have always been somewhat interested in science. My father has worked as a research scientist for longer than I have been alive, and hearing him talk about his work was always incredibly interesting for me. Working in research as an undergraduate has further solidified my passion for learning new information and contributing to new ideas and understandings.

**If you were able to give one piece of advice to your younger self, what would that be?**

**WOH:** Never assume that you're above messing something up in the lab, no matter how basic. If there's an inconsistency in your observations, don't let overconfidence in your competency misdirect you in explaining it. You'll probably solve the mystery faster that way.

**JRK:** I would definitely tell myself to enjoy the process more. Sometimes you can get so consumed by certain outcomes or expectations, that you miss how enjoyable the time you spend in the lab can be.

**Are there specific individuals or groups who have influenced your philosophy or approach to science?**

**WOH:** Outside of my mentors, nobody specific comes to mind. However, many people have shared mantras of sorts with me, and some really resonated. Absence of evidence is not evidence of absence. All models are wrong, but some are useful. A hypothesis is something that we try our hardest to disprove, and “truth” is just what we call it when we all fail miserably. I don't remember where I first heard any of them, but they cross my mind frequently.

**HRS:** My approach to research has been influenced by my mentor Debbie's advice to “follow the science.” While it's our default as researchers to use techniques we're familiar with, she has always encouraged me to take creative approaches, including entering collaborations, to pursue the problem at hand. This has allowed me to learn how to address thermodynamic, kinetic, cell-based, and transcriptomic questions. Although approaching problems from multiple scientific angles can often feel painstakingly slow and result in many failures, I have gained confidence in tackling difficult questions and feel that no technique is out of reach.

**What were the strongest aspects of your collaboration as co-first authors?**

**WOH:** I valued how freely data, ideas, and reagents flowed between us. Sometimes, collaborations can be a nightmare, where the project moving along is heavily rate-limited by information exchange. That certainly wasn't the case with this project, and all of us were readily up to date. Halley and Jackson were excellent partners in both experimentation and discussion.

**JRK:** Coming into this project as an undergraduate, I was certainly intimidated by the idea of collaborating with both a postdoctoral fellow and a graduate student. However, every interaction I have had with my collaborators and other lab members has been incredibly friendly and open. This made the project a very fun collaborative experience, that certainly made the process enjoyable.

**How did you decide to work together as co-first authors?**

**HRS:** In the fall of 2022, Wayne discovered that many nucleic acid–binding proteins engage in direct transfer, and I was wrapping up a paper characterizing ERα’s RNA-binding capabilities. Wayne reached out to me inquiring about a collaboration to see if ERα engaged in direct transfer between RNA and DNA. It was clearly fodder for a robust joint study, and we took off from there!
